# Approaches to sharing trial results with adult patients and the public: a qualitative interview and focus group study

**DOI:** 10.1186/s13063-026-09944-5

**Published:** 2026-08-18

**Authors:** Sanjana Choudhury, Charlotte Albury, Gary S. Collins, Jennifer de Beyer, Sally Hopewell

**Affiliations:** 1 Nuffield Department of Orthopaedics, Rheumatology and Musculoskeletal Sciences/Centre for Statistics in Medicine, University of Oxford, Oxford, UK; 2 Nuffield Department of Primary Care Health Sciences, University of Oxford, Oxford, UK; 3 Department of Applied Health Sciences, School of Health Sciences, College of Medicine and Health, University of Birmingham, Birmingham, UK; 4 National Institute for Health and care Research (NIHR) Biomedical Research Centre: Birmingham, University Hospitals Birmingham NHS Foundation Trust and University of Birmingham, Birmingham, UK

**Keywords:** Dissemination, Trial result, Stakeholder perspective, Qualitative research, Thematic analysis, Patient and public involvement, Barriers and facilitators

## Abstract

**Background:**

Sharing trial results with participants is increasingly recognised as an ethical obligation that fosters trust, respect, and engagement. Yet in practice, results are rarely shared. Researchers often lack guidance on what information to include or how best to present it. To create guidance that is both practical and meaningful, it is important to understand the perspectives of patient and public contributors and researchers. We explored these perspectives to inform the development of a reporting guideline for sharing trial results with patients and the public.

**Methods:**

We conducted a qualitative study using semi-structured interviews followed by focus group discussions. Participants included researchers with experience in clinical trials (such as chief investigators, trial managers, and statisticians), members of the public, and patient representatives. We conducted 29 interviews and 3 focus groups between May and November 2024. Participants were invited to describe, in their own words, what information they considered essential or optional in trial result summaries and how it should be communicated. Data were analysed using the Framework Method, informed by constant comparison to develop themes, with coding and interpretation supported by both the research team and participants.

**Results:**

We identified six descriptive themes that reflected the participants’ experiences and perspectives. Participants strongly endorsed the principle of sharing trial results with patients and the public, citing respect, transparency, and empowerment as key reasons. Researchers highlighted uncertainties about what to report, when to disseminate, and how to present results, and noted resource and structural barriers. Patients and public participants prioritised plain language, clarity, and contextualisation, and stressed the need for inclusive communication for underrepresented groups. There was broad agreement on core elements for trial result summaries, including study purpose, key findings, potential harms, implications, and next steps. Preferences varied, however, regarding the level of detail and presentation style. Flexibility, with a set of core items reported consistently and complemented by optional elements where relevant, was seen as the most workable solution.

**Conclusions:**

This study highlights how patients, members of the public, and researchers view the sharing of clinical trial results as both a moral responsibility and a practical challenge. The findings provide evidence-based recommendations to guide the development of a reporting guideline that supports consistent, transparent, and meaningful communication of trial results beyond the research community.

**Supplementary Information:**

The online version contains supplementary material available at https://doi.org/10.1186/s13063-026-09944-5.

## Background and rationale

Researchers and participants value the opportunity to share and receive study results [1–3]. Sharing study results fosters participant trust and engagement, increases their sense of ownership, and is widely regarded as a basic right [4, 5]. The UK’s recent Medicines for Human Use (Clinical Trials) (Amendment) Regulations 2025 require trial sponsors of investigational medicinal products to offer participants a summary of results within 12 months of trial completion [6].

Despite increasing policy attention, sharing trial results with participants is now more common but remains inconsistent. A study that surveyed authors of 1818 clinical trials indexed in PubMed in 2014–2015 reported that only 27% shared results with participants [7]. Only ~ 2% of clinical trials completed worldwide between 2015 and 2017 shared a plain language summary of results with participants [8]. More recently, the latest UK Health Research Authority (HRA) transparency report found that 52% of UK trials completed in 2023 had shared, or were in the process of sharing, results with participants [9]. A 2025 systematic review highlighted that even among investigators who plan to share trial data, 81% rely entirely on passive dissemination methods that require participants to independently locate the information [10]. Across Europe, only 14% of trials between 1 January 2017 and 30 June 2020 successfully provided plain language summaries (PLSs) within the public domain, and these remained difficult to find via standard search engines [11]. Even when summaries are shared with participants, in a sample of NIHR-funded trials published between 2016 and 2021, over 85% were flagged as difficult to read [12].

Previous work has highlighted participants' preferences for receiving results and barriers to sharing trial results [13]. Researchers are often uncertain about what to communicate, the most appropriate format, and the timing of dissemination. These challenges are compounded by the absence of clear guidance, communication support or institutional processes to support reporting of trial results [7, 13–15]. The result is considerable variation in practice [13].

While a reporting guideline alone would not address all organisational and resource-related barriers, it could provide a structured framework to support more consistent and transparent communication of trial results. However, our recent scoping review found no such reporting guideline [16]. The closest available resource is the guidance produced by the RECAP programme, which identified broad principles relating to what, when, how, and by whom trial results should be communicated, but did not offer a structured itemised guideline [17].

Developing a reporting guideline requires a comprehensive list of candidate reporting items. Similar to RECAP, our programme of work drew on a scoping review to identify existing recommendations [16]. However, we also sought to generate new reporting items directly from patient/public contributors and trial professionals by exploring their experiences and perspectives. The findings subsequently informed the generation of candidate items for the Delphi phase of the reporting guideline development process, which was conducted separately after this study [18].

We also explored stakeholder perspectives and preferences on communication strategies, barriers to implementation and areas of improvement for sharing trial results with patients and the public. The findings were intended to inform the development of a reporting guideline for communicating trial results in ways that are clear, accessible, and meaningful, while also identifying wider organisational and resource-related barriers that may influence implementation.

Although trial participants are the primary audience for many trial result summaries, these summaries are often made available more broadly to patients, patient representatives, and members of the public. We therefore considered perspectives on communicating trial results beyond trial participants alone. Where discussing the ethical rationale for result sharing, we refer specifically to trial participants; where discussing dissemination materials and reporting guidance, we refer more broadly to patients and the public.

Given the ongoing regulatory developments and policy focus within the UK, this study was conducted primarily with UK-based stakeholders to explore perspectives on sharing trial results within the context of the UK research environment.

## Methods

### Protocol and registration

We registered the study protocol on Open Science Framework (OSF) [19]. Reporting follows the “Standards for Reporting Qualitative Research” (SRQR)* [20] (Additional file 1). The study is underpinned by a pragmatic approach that focuses on practicality and usefulness (not only “what is” but also “what might be”). The aim was to gather insights on how to share trial results with patients and the public and identify solutions to inform the development of a reporting guideline for these results. The study was approved by the University of Oxford Medical Sciences Interdivisional Research Ethics Committee [reference R93417/RE001].

Our study adopted an open-ended qualitative approach using semi-structured interviews, giving people the space and time to share their thoughts without influencing them with a predefined list of candidate items. Participants were asked directly what information they considered essential or optional in a trial results summary, allowing candidate reporting items to emerge inductively from their own experiences and priorities. To broaden and refine these findings, we subsequently held three focus groups to explore the emerging reporting items, communication strategies and implementation issues.

### Sampling and participants

Participants were eligible if they were aged 18 years or older, able and willing to provide verbal informed consent, and identified as belonging to one of two stakeholder groups: *Group 1*—researchers working in trials or *Group 2*—members of the public and patient representatives.

We used a mix of purposive and snowball sampling to recruit participants. Researchers with a range of clinical trial-related roles, including trial managers, chief investigators, statisticians, clinicians, researchers with expertise in clinical trial methodology and other trial professionals, were targeted, recognising that responsibility for result sharing is often unclear. We aimed to ensure representation across roles, with at least one participant from each category and participants representing at least two institutions.

Participants in the public and patient group were recruited from across the UK, to reflect diversity in age, gender, ethnicity, language, socioeconomic background, and lived health conditions. Particular emphasis was placed on including underrepresented and marginalised communities, such as ethnic minority groups and those affiliated with different religious communities. To support recruitment, we advertised through relevant networks such as patient and public involvement (PPI) networks, clinical trial networks (e.g. the UK Trial Managers’ Network), local communities, religious groups (e.g. the NIHR Diversity in Research Network, Islamic Society of Women in Oxford), and social media (LinkedIn, X). (Additional file 2) Snowball sampling was also used, with participants identified through mutual contacts and established networks.

Based on the considerations of the five aspects of information power (scope of the study, sample specificity, theoretical framework, expected quality of dialogue, planned analytical approach) and evidence from similar studies, we aimed to recruit 10 to 15 individuals per stakeholder group [21, 22]. Data collection and analysis were conducted iteratively, and recruitment continued until the research team judged that adequate information power was achieved for the emerging themes.

### Data collection

We conducted 29 semi-structured interviews between 30 May and 22 October 2024. The interview topic guide was informed by existing literature and gaps identified in our scoping review and was refined following feedback and piloting with researchers and PPI representatives to ensure alignment with the study objectives [16]. The guide was updated iteratively as new topics arose (Additional file 3). Questions were phrased in the format “What items would you like to see in the trial results report? And why?” or “What are the mandatory/optional items?”; explicit items were not provided in the questions.

Interviews lasted around 45 min on average and were conducted in English, either face-to-face, by telephone, or online via Microsoft Teams, depending on participant preference. Arrangements were made via email, where eligibility was confirmed, and a participant information sheet was provided [19]. SC conducted all interviews, which were recorded by Microsoft Teams or encrypted Dictaphones and transcribed verbatim. Verbal consent was obtained at the start of each interview.

We also held three online focus groups on 25 October, 1 November, and 5 November 2024. Each session lasted approximately 1 h and included a mix of researchers and public and patient participants. All focus groups were facilitated by SC using a semi-structured topic guide (Additional file 3). Preliminary findings from the interview transcript analysis were shared during the focus groups to support validation of the emerging analysis and to elicit additional perspectives.

### Data analysis

We used a combined analytical approach, drawing on the structured organisation of the Framework Method by Gale et al. and the iterative and theory-generating processes of constant comparison by Corbin and Strauss [23, 24]. We followed the seven stages of the Framework Method: (1) transcription, (2) familiarisation, (3) coding, (4) developing a working analytical framework, (5) applying the analytical framework, (6) charting data into the framework matrix, and (7) interpreting data [23].

All transcripts were imported into NVivo 12 for coding. Both deductive (based on the topic guide) and inductive (participant-led) approaches were used, with in vivo codes applied where participants’ own words captured concepts most appropriately. Initial coding of the first five transcripts informed the development of a working analytical framework, which was then applied across all transcripts. Codes were organised into categories, and axial coding was used to explore connections between them. Through constant comparison within and across transcripts, six descriptive themes were developed to represent the findings [25].

As the purpose of this study was to identify candidate reporting items and implementation considerations for subsequent reporting guideline development, data from researchers, patient representatives, and members of the public were analysed together rather than compared between stakeholder groups.

### Reflexivity and data quality

We integrated reflexivity in all stages of the study by ongoing self-reflection of researcher subjectivities and positionalities [26, 27]. At the preliminary stage, a patient and public panel reviewed the study plan and the topic guide. Measures were taken to broaden the diversity of participants in terms of age, gender, socioeconomic background, lived conditions, and previous trial experiences. During interviews, demographic questions were placed at the end to give participants more control, and venues were chosen according to their preference (home, workplace, or virtual). SC positioned herself as a “guest”, adopting a back-seat role that allowed participants to set the pace and fostered authentic dialogue. This approach facilitated rapport and generated high-quality, in-depth data.

SC maintained a diary to note down reflections and thoughts throughout the research process. The coding stages were discussed extensively by the research team while we continuously interrogated our own biases, subjectivities, and positionality, ensuring self-awareness and critical reflection. The research team brought complementary expertise to the study. CA has extensive experience in qualitative health research, while SH, GSC, and JDB bring complementary expertise in clinical trials, research methodology, mixed-methods research, and reporting guidelines. This combination of perspectives supported reflexivity by enabling SC to reflect on how her own perspectives and experiences may have shaped the analysis and interpretation of the data.

Thematic findings from interviews were shared with focus group participants for validation and refinement. Participants reflected on the identified themes and commented on their relevance and accuracy in relation to their own experiences. Comparing interview and focus group data enabled us to examine whether similar patterns emerged across diverse participants. This process not only enhanced the robustness of the analysis but also ensured that participants’ perspectives remained central to interpretation, thereby increasing confidence in the findings.

## Results

### Characteristics of participants

We interviewed *29 participants* (Group 1: researchers working in trials, *n* = 15; Group 2: members of the public and patient representatives, *n* = 14), primarily from the United Kingdom, with one participant from Ireland. We now refer to them as our interviewees, as the term participants is used extensively to refer to trial participants. A summary of interviewee characteristics is provided in Additional file 4, with individual characteristics presented in Tables 1a and b.

**Table 1:** Characteristics of individual interviewees: (a) Group 1 and (b) Group 2

(a) **Group** **1**:** researchers working in trials**					
**ID**	**Ethnicity**	**Age range**	**Sex**	**Current profession**	
A1	White-British	26–35	Female	Senior medical writer	
A2	White-British	36–45	Female	Clinical trial monitor lead	
A3	White-British	18–25	Female	Clinical trial coordinator	
A4	White-British	56–65	Male	Principal medical writer	
A5	White-British	46–55	Female	Study delivery officer	
A6	White-Scottish	46–55	Female	Senior trial manager	
A7	White-British	26–35	Female	Junior qualitative researcher	
A8	White-British	56–65	Female	Intensive care research nurse	
A9	White other-Turkish	26–35	Female	Clinical trial manager	
A10	White-British	36–45	Male	Chief-investigator in clinical trials	
A11	White-English	36–45	Male	Professor of clinical trials	
A12	White-American	36–45	Female	PhD student in clinical trials methodology	
A13	White-British	46–55	Female	Research trial manager	
A14	White-British	36–45	Male	Professor of Clinical trials & medical statistics	
A15	Asian-Indian	46–55	Female	Senior researcher	
(b) **Group 2**: **members of the public and patient representatives**					
**ID**	**Ethnicity**	**Age range**	**Sex**	**Native language**	**Representative/special interest**
B1	White-British	> 65	Female	English	PPI representative
B2	White-British	> 65	Male	English	Dementia
B3	White-British	46–55	Female	English	Autism, Long-term physical and mental health conditions patient
B4	White-Welsh	> 65	Male	English	Dementia
B5	White-British	> 65	Female	English	Diabetes
B6	White-British	56–65	Female	English	Oesophageal cancer survivor
B7	White-British	56–65	Female	English	Psychology
B8	White-British	56–65	Male	English	Member of the public
B9	White-British	18–25	Male	English	Autism, Identifies as LGBTQ +
B10	White-British	18–25	Female	English	Member of the public
B11	Asian-Pakistani	36–45	Female	Urdu	Member of the public
B12	Asian-Indian	36–45	Female	Bengali	Member of the public
B13	Asian-Pakistani	26–35	Female	Urdu	Member of the public
B14	Black-African	18–25	Female	English	Member of the public

Overall, 72.4% of interviewees were female and 75.9% identified as White British. Interviewees from both groups represented a range of ethnic backgrounds, including Asian and Black ethnicities, which were widely distributed across age groups.

### Sharing trial results with patients and the public

Researchers noted that in non-commercial settings, increased funding and a growing emphasis on PPI, especially after the COVID-19 pandemic, have helped raise awareness of the value of patient and public involvement in research. However, result sharing is not routine, and researchers often rely on personal judgment when deciding whether and how trial results should be shared with patients and the public.

In Group 1 (*n* = 15), four interviewees said they had shared trial results, three planned to do so, and two had no plans for dissemination. The formats used to share results included a lay summary, plain language abstract, short video or newsletter, and public dissemination event.

Dissemination planning varied: two interviewees followed the European Union Clinical Trials Regulation (EU CTR) for trial result sharing [28]. The EU CTR requires sponsors of clinical trials of investigational medicinal products to submit a lay summary of trial results to the Clinical Trials Information System (CTIS) within specified timelines [28]. Four interviewees were at an early stage with no detailed plan, while two had established detailed dissemination strategies. Five interviewees reported having a PPI group in their trials.

Within Group 2 (*n* = 14), six interviewees had previous experience of taking part in research, including three in clinical trials. Only one interviewee had received results directly, while five received no feedback. Ten interviewees had experience of PPI activities, but only three had ever seen trial results, mostly as research papers.

We developed six descriptive themes to discuss our results:

1. No participants no trials—it is all about respect and recognition;
2. Results, clinical implications and demography are most meaningful items to report when sharing trial results;
3. Clarity, accessibility and respect for the reader are important when presenting information;
4. No “one size fits all”—communication strategy must be tailored;
5. Barriers to sharing trial results;
6. Areas of improvement for sharing trial results.

**Fig. 1:**
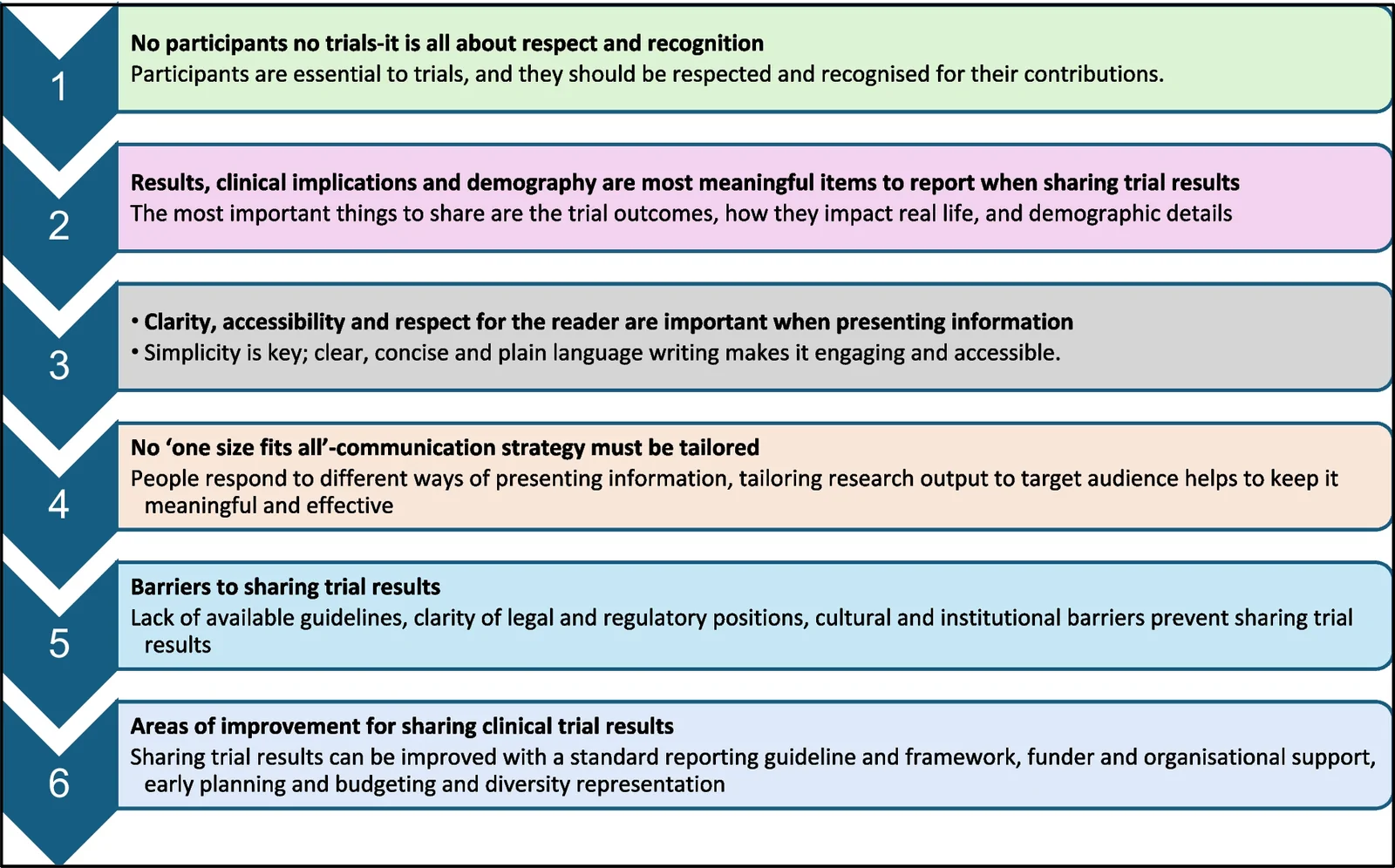
A summary of the overarching themes

A brief overview of these themes is shown in Fig. 1.

#### Theme 1: “no participants no trials”—it is all about respect and recognition

Interviewees from both groups expressed several reasons for sharing trial results, which centred on the importance of respecting and recognising participants for their involvement and the ethical and moral responsibility of researchers to share results. Many stated that sharing results could motivate future participation, foster trust in research, and help people make informed health decisions.

“But I think if people have committed, agreed to commit time to it, then it makes them feel a bit more special, makes them feel heard.” (B1)

##### Sharing negative results

Interviewees from both groups unanimously agreed that all results should be shared, whether positive or negative. There is value in learning from failure and it is important for transparency and to reduce research waste.

Group 1 interviewees noted that how results are presented matters, even if the trial was not successful.

“If somebody’s taking part in research and you’ve had a negative trial, they might be a bit disappointed, but hopefully you could word your lay summary in a way that explains to them that even though the result hasn’t shown anything, it has a way because it means we know not to keep repeating the same mistakes or to do that again”. (A6).

Researchers should be mindful that trial results may be emotionally difficult for some individuals, and interviewees suggested that PPI contributors could help inform appropriate approaches to communication and support. Rather than assuming that all participants would want to receive results, interviewees emphasised the importance of offering individuals a choice about whether they wished to access the information.

“Here the results are available if you would like to look … Here is a way to get to them if you're interested.” (A2)

#### Theme 2: results, clinical implications, and demography are the most meaningful items to report when sharing trial results

Interviewees from both groups most frequently identified trial results and their clinical implications as information that should be included in a results report. They wanted to understand the key message, what the study found, whether the intervention was beneficial, harmful, or showed no meaningful difference, and how the findings might improve a condition or quality of life. Many were also interested in whether the results might influence governmental policy. Demographic information was also frequently mentioned, as interviewees wanted to judge whether the results were relevant to them. Many were curious about the trial’s future direction: will there be a next phase, is there an opportunity to participate, and what is planned for the findings?

Another commonly mentioned item was a brief overview of the trial’s aim and background. This is perhaps particularly important for long-running trials as participants may have forgotten details from the participant information sheet by the time they see the results. Having the aim and background would also help put things into perspective for members of the public.

However, the most frequently mentioned items may not always be the most important. As Saldana suggests, is there any value in measuring a “qualitative mean”, and does it truly enhance the analysis? [29] For example, few interviewees mentioned thanking trial participants as an important item. Did they perhaps not view this item as important, or did they view saying thank you to be an automatic or expected gesture that does not need explicit mention or reinforcement:

“Well, that’s a given, isn’t it…You know you always say thank you. I mean simple 2 words. Simplest word”. (B2).

When asked to distinguish between information that should be essential and information that could be optional, interviewees across both stakeholder groups recognised that not all items would be equally important in every context. While there was broad agreement on a core set of information that should be included in all trial result summaries, interviewees also felt that additional information could be included where relevant or where readers wished to access more detail. This was viewed as a practical way of balancing completeness with accessibility and accommodating different information needs.

In Table 2, we present a full list of items (non-ranked) suggested by interviewees, with supporting quotes. By including less frequently mentioned items, we hope to provide valuable depth and nuance. The items presented in Table 2 were derived inductively from interviewee suggestions from both groups. Similar or overlapping items were grouped together and duplicate suggestions were removed. The resulting categories remained close to interviewees’ original descriptions and were intended to provide a descriptive summary of the information they considered meaningful to include in trial result summaries.

**Table 2:** Items suggested by interviewees (Group 1 and 2) for inclusion in trial result summaries

Categories	Illustrative quotes
1 Results	*“What are the findings? I think that would be a key one.” (B7)*
2 Clinical implications	*“The significance of the results from the trial. And how they might be implemented on the standard of care. “(A3)*
3 Improvement of quality of life	*“It might also be very useful from a patient perspective to if the clinical trial that involve quality of life and things like that to see if we can present some results on that as well, and how being on the trial has affected quality.” (A3)*
4 Changes to NHS policy	*“I guess implications for the NHS for our trials”. (A8)*
5 Economic implication	*“I guess cost implications for the NHS maybe also interesting for the public as well.” (A8)*
6 Comparison with other research	*“How it compares to what's known from before, you know other research as well.” (A15)*
7 Generalisability	*“I'd be interested to know how the sample met, because I know from the work, I do that that this that samples are often not reflective of the of the population, let alone the population with diabetes.” (B5)*
8 Demography	*“Interested in the demographics of the people involved, you know the other participants”. (B4)*
9 Number of participants	*“How many people took part?” (A5)*
10 Aim and background	*“So, the purpose like why it was being done, yeah. “(B14)*
11 Future direction	*“what's the next stage say from the outcome of this? Is there a paper being written to recommend a change in standard care? “(A2)*
12 Next steps	*“What are the next steps going forward and how's my time gonna be?” (B9)*
13 Milestones (patient journey)	*“To make it interesting, maybe something about the people involved* *You know the, the people, the researchers and some of the difficulties maybe they had and how they overcame them and make it a bit more of a story. But you know, people like a story, don't they?” (B1)*
14 Further information	*“And then their contact details if they need…If they would like more information or if they would like to discuss it with somebody else that that would be handy as well.” (A5)*
15 Publication details	*“Like it's…published or it is accessible for all the people like how can the normal people read that outcome?” (B11)*
16 Side effects	*“I guess if there were some serious adverse effects…. worth knowing. I mean, I'm yeah, I tend to want the good news ha-ha.” (B8)*
17 Thank you	*“I definitely want to feel like my input was valued and like my time was respected…definitely like a thank you for the people that are actually involved- would be great because I think a lot of the time that's kind of forgotten.” (B9)*
18 Strengths and limitations of the findings	*“So, I think there should be accessible communication about the strengths and limitations of findings. What can we be more sure of? What are we less sure of, regarding the findings? What are the certainties? What is the uncertainty?” (B3)*
19 How to talk with healthcare providers about the results	*“Yeah, I think using a simple English as possible and very clear, so that people can decide whether how relevant it is to them and then also like referencing it back to talk to your GP if you have any further questions. So just like usual things.” (B13)*
20 Logistics	*“I've not specifically said it, but you would usually say who funded it…You would want to mention the Institute and maybe the CI name as well.” (A6)*
21 Methodology	*“Methodology-I'd be interested in, especially if it could be summarised.” (B10)*
22 What could be done better	*“I think it's really important to be honest about… What could have we done better in the trial?” (B9)*
23 Ethics approval details	*“Uh, yes, I would expect to see that very much…I'd want to have that reassurance that ethically this has been approved.” (B7)*
24 Frequently asked questions	*“Maybe there's like a frequently asked questions…. that could be interesting because eventually people might still be asking in that in the future after the trial, so, that might be interesting.” (A9)*

#### Theme 3: clarity, accessibility, and respect for the reader are important when presenting information

Interviewees shared a range of views on how information should be best presented, emphasising clarity, accessibility, and respect for the reader. Researchers highlighted the importance of using appropriate and respectful language, whereas patients and members of the public prioritised plain language, brevity, and empathy.

##### Subtheme: simplicity is key

This subtheme relates to the principle of “less is more”, highlighting the value of clear and concise communication. Academic writing is often inaccessible and intimidating. When the target audience is the general public, a balance must be struck between sufficient detail and clarity:

“I think it needs to be easily accessible so that you can just flick to it straight away”. (B5)

Patients and members of the public favoured short, jargon-free sections with clear headings, executive summaries, and white space. They valued brevity but warned against oversimplification. Some expressed feeling insulted when information was oversimplified, emphasising that they, as laypeople, should be treated as capable of understanding complex concepts:

“Just because we are lay people and people on the street, we we’ve still got brains in our heads, and we can work things out”. (B2)

##### Balanced use of visuals

Responses varied when it came to visuals as the level of interpretation varies between people. Some found graphs and infographics helpful, while others preferred text or worried that visuals might clutter or reduce professionalism. High-quality infographics were seen as a good compromise but potentially costly:

“So where possible you communicate what you can visually with infographics, then you use text for the parts you can’t”. (B3)

##### PPI review

Interviewees shared that PPI partners can offer unique perspectives during the result-sharing process, often identifying the most impactful and practical information to include, helping to keep the content focused and useful. However, PPI involvement should not only be during final-stage review. It should be a continuous part of the process, integrated from the very start.

##### Translations

Translation was viewed as important to reduce health inequalities, with suggestions for written, audio, and video formats. Cost and quality of translations were noted as challenges.

Researchers discussed whether the value of translation justifies the cost. One researcher noted that much of the research contact is usually in English and questioned the impact of translating only some documents.

##### Length and timing

Preferences for the length of the summary varied, but flexibility was considered key. Interviewees cautioned that people with learning differences may find lengthy documents difficult to read:

“I struggle with things that are very long to read because of my autism and my ADHD”. (B9)

Researchers also raised concerns about the potential for misunderstanding when more information is provided:

“I do suppose it is slightly double edged. More information that leads to more potential of misunderstanding or raising concerns”. (A14)

Researchers often felt a sense of urgency to share results while fresh and felt that waiting too long could reduce enthusiasm. However, patients and members of the public had mixed opinions: some wanted to be the first to know the results, while others valued peer review for its credibility. A possible solution is simultaneous sharing—releasing results to patients and the public at the same time as academic publication—although this can be challenging as journals may impose embargoes or funders may have confidentiality agreements in place. In commercial trials, proprietary concerns also add complexity.

Regardless, it was considered essential for trial participants to have a clear expectation of when they would receive results.

#### Theme 4: no “one size fits all”—communication strategy must be tailored

Interviewees emphasised that people respond to different ways of presenting information and that there is no one-size-fits-all approach. Preferences depend on personal learning styles, age, culture, and access to technology. As one interviewee shared:

“British people over here they maybe… they like to read…But in Asian countries…. it’s my personal [opinion] that they want to watch or audio [hear]”. (B12)

##### Formats and their trade-offs

Interviewees discussed a range of formats, noting that each had distinct benefits and limitations:

- Audio: valued by people with visual impairments or low literacy. However, some found phone calls intrusive, worried about accents and clarity, and felt calls could be easily missed.
- Digital media (emails, PDFs, videos): praised for being convenient, mobile-friendly, and environmentally friendly, However, some raised concerns about digital exclusion for those without internet access or devices. Videos drew mixed reactions: some welcomed them as engaging, while others preferred written reports.

“I think like sustainability wise an e-mail makes more sense”. (B10)

- Social media: recognised as a powerful tool for engagement and reach, though interviewees worried about credibility, navigation difficulties, and data privacy issues:

“I’m on the fence with social media”. (B2)

- Interactive sessions (webinars, Q&A events, podcasts): encouraged two-way communication and trust through clinician involvement, but posed challenges around affordability, scheduling, and anxiety for some interviewees.

Offering choice was seen as the ideal solution, allowing individuals to select the formats that work best for them.

#### Theme 5: barriers to sharing trial results

Group 1 interviewees (researchers) identified a wide range of barriers that complicate or delay the sharing of trial results with patients and the public.

##### Lack of guidance and consistency

Interviewees felt that the absence of clear guidance poses a major challenge to sharing. Terminology such as “lay summary”, “plain language summary”, and “patient summary” are used inconsistently, creating confusion. Clear standardised templates and guidance are not available to standardise language and practices across the field.

“We just worked as a group. We weren't following any guidance, to my recollection about it, to be honest.” (A14)

##### Legal and regulatory barriers

Legal requirements to share results are limited, particularly in the UK and USA, where dissemination to participants has historically not been mandated. However, this is beginning to change in the UK. Under the *Medicines for Human Use (Clinical Trials) (Amendment) Regulations 2025*, trial sponsors will be required, from 28 April 2026, to offer a summary of trial results of investigational medicinal products to participants within 12 months of trial completion [6].

Industry trialists worried that sharing results could be misconstrued as marketing. Embargoes from journals or funders, intellectual property concerns, and data protection restrictions (e.g. reliance on NHS-only software) further complicate timely dissemination.

##### Practical and logistical barriers

Resource constraints were a recurring concern, particularly for academic and non-commercial trials with limited budgets for participant engagement. Large trials faced the additional challenge of disseminating results to thousands of participants. Staff turnover in long-running studies can also disrupt continuity.

##### Cultural and institutional barriers

Researchers noted that lay summaries were often treated as a low priority at an institutional level. The skills, time, and expertise needed to produce high-quality lay summaries were often underestimated, with limited training or support provided. At an individual level, some interviewees described a lack of confidence in writing plain-language summaries, recognising that this requires specific communication skills to avoid misinterpretation.

##### Communication and engagement barriers

Translating complex data into an accessible lay summary was seen as challenging. Researchers observed that trial results rarely feature in mainstream media, while digital literacy barriers limit access for some groups. Trials face competition for attention from more engaging media on participants’ devices, and results can feel disconnected from everyday concerns.

##### Timing and continuity barriers

Delays in disseminating results were felt to undermine momentum, reducing motivation for research teams and weakening participants’ sense of connection to the study:

“Publications will take months, years, sometimes, so it’d be really hard for me as a clinician to say, well, I know the results, but I can’t tell you until this arbitrary process is done. Even though these results are directly related to you”. (A12)

#### Theme 6: areas of improvement for sharing trial results

Interviewees identified several opportunities to strengthen how trial results are communicated.

##### Guideline availability and awareness

Interviewees called for regulatory bodies to standardise terminology for lay summaries and offer consistent templates. Industry writers shared that they typically followed EU CTR Article 537 and shared their awareness of the “Good Lay Summary Practice” (GLSP) guideline [30]. However, they noted that while GLSP provides structure, it is lengthy and lacks practical advice with limited inclusivity.

In contrast, NHS and non-commercial researchers shared that they were often unaware of formal guidelines for creating lay summaries, instead relying on personal judgment. A standardised reporting guideline with an evolving checklist was widely supported as a potential solution that could address many of the challenges in preparing a lay summary for clinical trials.

##### Funders and organisational support

Researchers noted that funders are now prioritising results dissemination, which is acting as fuel for academic, non-commercial trials to share results with their participants:

“Having clear guidance from regulators about what’s acceptable and what’s not”. (A1)

Interviewees suggested that journals could contribute to dissemination efforts by mandating a public engagement section.

##### Early planning and budgeting

Interviewees suggested that dissemination be planned from the start of the trial and built into the protocol and funding application. Budgets should cover PPI involvement, plain language writing, design, technology, legal compliance, and maintaining participant contact details. Dissemination roles should be defined from the start, with chief investigators supported by trial managers and coordinators. Interviewees believed that PPI involvement is essential to ensure results are presented clearly and respectfully.

Interviewees noted that in most trials, results are shared with participants through local hospital teams rather than the central trial team. They suggested that dissemination be discussed during site initiation visits and included in contracts so that local teams are aware of and committed to delivering their responsibilities from the outset. Interviewees suggested that clinical trials units tailor documents for local sites to ease distribution, as local teams often lack time or resources for active involvement in dissemination.

After sharing the summaries, trial teams should also check how effective they were by asking patients for feedback.

“That’s something we’re missing… is finding out what is the impact of that? what’s the best way of doing that to make sure that it has the greatest reach to the people that need to see it?” (A12)

##### Aligning results reports with participant-facing materials

Interviewees noted that results reports should mirror the language and terminology used in the participant information sheet, which trial participants are already familiar with. Including a statement in consent forms about whether and how results will be shared was seen as a way to ensure accountability.

##### Diversity and representation

Diversity in research is essential for meaningful outcomes. Trust issues, rooted in societal inequalities such as anti-Blackness, anti-immigrant sentiment, and Islamophobia, often prevent participation. Interviewees called for inclusive trial design, recruitment strategies tailored to underrepresented groups, and staff diversity to build trust. Accessibility must go beyond physical barriers, recognising invisible disabilities and the intersectionality of marginalised identities. Creating safe, welcoming environments where all participants feel valued was seen as key to genuine engagement. In the words of B9:

“I'm also someone from that (Autistic) community… “It's about kind of the environment and the atmosphere that's created and just which I think was done really well.

And I think that kind of gave me the opportunity to contribute because it's kind of the first time I've done anything like this because I've always thought like, I don't know if my opinions really going to be valued…You kind of allowed that time and that space to become more accessible. And I think that's what kind of encouraged me to stay and not just feel completely overwhelmed straight away. So, I'd just like to say thank you for that”.

##### Identifying and reaching the audience

Interviewees urged researchers to consider who needs the results most, including patients, carers, clinicians, policymakers, family members, and charities:

“But it's also thinking well, who's got most to gain. And who are the hardest to reach? And can we try to get the easy to reach people, but also the harder to reach people as well equally”. (A11)

Interviewees cautioned that an additional layer of complexity arises around sharing results with family members if the trial participant has passed away, which is more likely in certain trials, such as those related to cancer, palliative care, or intensive care. Determining how to handle this ethically and compassionately is challenging: should consent be obtained beforehand, and if so, who gets to make that decision? These questions require careful consideration, balancing respect for patient privacy with the needs of grieving families who may find closure or insight in the trial’s findings.

##### Engaging the public

Interviewees mentioned that trial evidence is not part of mainstream conversation and is often overshadowed by sensational posts on social media:

“If, like, the voice from the trial is overshadowed by more sensational stuff on TikTok, then it’s and it’s not in that space, then that’s a problem, because people…they wouldn’t read the paper. They wouldn’t get the message.” (B13)

Interviewees encouraged researchers to use social media creatively and professionally, partner with charities, and host community events to improve reach. Dedicated trial websites and dashboards were seen as helpful resources, with the caveat that they require maintenance and good search visibility to be effective.

## Discussion

This study used semi-structured interviews and focus groups with researchers, patients, and members of the public to gather their opinions on the importance of sharing trial results with patients and the public, priorities for reporting, preferences for communication, barriers to implementation, and potential areas of improvement.

Interviewees identified what information should be shared and how it should be presented. The reporting priorities identified in our interviews were broadly consistent with those reported in previous studies of participants' preferences for trial result summaries and with the results of our prior scoping review [16, 17]. Interviewees also highlighted additional reporting items that were rarely or not represented in published guidance identified through our previous scoping review, including items such as clinical implications, comparison with existing research, economic implications, changes to healthcare policy, strengths and limitations, and milestones in the patient journey. Unlike most guidance development approaches identified in our scoping review, which asked stakeholders to evaluate or rank predefined reporting items, our open-ended qualitative approach allowed participants to generate reporting items inductively from their own experiences and priorities. This suggests that open-ended qualitative inquiry can identify reporting items that are important to intended audiences but may not emerge through approaches based on predefined candidate items. These additional items were subsequently taken forward into the Delphi survey for further evaluation, which was conducted separately after this study [18].

Interviewees highlighted the importance of sharing results in ways that are meaningful, understandable, and respectful of participants’ contributions to research. Previous studies have noted that concerns about the psychological impact of sharing negative trial results can act as a barrier to sharing results [2]. Despite these concerns, our patient and public interviewees overwhelmingly expressed a desire to receive the results regardless of outcome. They emphasised that the nature of trials involves uncertainty, and that trial participants understand and accept this fact as long as the results are shared sensitively. Similar findings were reported in recent literature, where participants described result sharing as important for trust, transparency, and feeling valued [10, 13, 17].

While preparing lay summaries is an essential step, interviewees noted that their usefulness must also be evaluated. Seeking feedback from patients and the public after summaries are shared can help determine whether the intended goals of clarity, understanding, and trust have been met, and guide improvements in future dissemination efforts.

Interviewees emphasised the importance of inclusive reporting and recruitment. Both this study and our earlier scoping review highlighted the lack of guidance on cultural and linguistic diversity in trial result dissemination [16]. Addressing this gap is essential to ensure that results reach, and are meaningful to, diverse communities.

Interviewees expressed a preference for flexibility in how trial results are communicated, recognising that individuals differ in their information needs and preferred modes of communication. While offering multiple dissemination formats may improve accessibility and engagement, it also has practical implications for trial teams in terms of time, resources, and infrastructure. A layered communication model, where all participants receive a concise summary of the key findings with opportunities to access additional information if desired, may provide a practical compromise.

These findings informed the development of candidate reporting items, distinguishing between core items that should be routinely reported and additional items that can be adapted according to context and audience. The candidate reporting items identified in Table 2 were taken forward for evaluation through a Delphi consensus process [18], while interviewees’ communication preferences informed the accompanying Explanation and Elaboration (E&E) guidance.

Recent improvements in result-sharing practices within the UK may reflect increasing policy attention to transparency and participant engagement. Nevertheless, similar to previous work, interviewees continued to describe practical barriers such as limited time, resources, uncertainty regarding responsibility for sharing results, and lack of organisational support, suggesting that regulatory requirements alone are unlikely to be sufficient [10, 17]. Similar to Bagita-Vangana et al., we found that ethical commitment alone is often insufficient to ensure dissemination in practice [10]. Practical guidance and organisational support are also needed if sharing results is to become routine practice.

### Strengths and limitations

Reporting guidance is usually based on expert opinion and the literature, and only seeks the opinions of those who will be affected by the guidance in selecting items from an existing list or in approving or improving existing wording. Here, we added an additional element to the evidence we will use for our reporting guideline by seeking the current opinions of the people who will be affected by the guidance before beginning to develop the guidance. We therefore have greater confidence that the items we have discovered in the literature are not outdated and do reflect current circumstances. We also guarded against missing key perspectives through the nature of inclusion and exclusion criteria for scoping or systematic reviews.

A key strength of this study was the inclusion of interviewees from a range of demographic and experiential backgrounds, with 24% identifying as non-White British and representation across socioeconomic backgrounds, lived conditions, age groups, and trial-related roles. Purposeful engagement with minority and community groups supported recruitment from a broad range of backgrounds.

Mixed focus groups involving both professionals and public contributors provided additional insight into how perspectives were negotiated through group discussion, allowing for insights that might not have been captured through individual interviews alone [31].

This study has several limitations. The absence of funding for participant reimbursement may have limited participation. Most patient and public participants had lived experience of chronic or long-term health conditions; therefore, the findings may not reflect the perspectives of individuals participating in trials related to acute conditions. As a result, the findings may not transfer directly to all clinical contexts, and further research in other patient groups would be valuable. We also did not collect detailed information on interviewees’ experience with specific trial types, such as trials of investigational medicinal products (CTIMPs), and were therefore unable to explore whether views differed according to regulatory context.

Interviewees were based in the UK, and their views may have been influenced by the UK healthcare and research context. Consequently, some findings may not transfer directly to settings with different regulatory requirements, research infrastructures, or cultural expectations. Recognising this limitation, subsequent stages of the reporting guideline development programme recruited participants from 16 countries through the Delphi survey to evaluate the candidate reporting items internationally [18]. As with all qualitative research, reflexivity and triangulation were used to minimise bias, although researcher subjectivity cannot be eliminated entirely.

## Conclusion

The study provides empirical evidence to inform the development of a reporting guideline for sharing trial results with patients and the public. While guidance alone will not address all barriers to dissemination, it may support more consistent and accessible communication of trial findings. Supporting researchers to share results clearly and appropriately may help bridge the gap between policy expectations and current practice.

## Supplementary Information

Supplementary Material 1.

## Data Availability

The de-identified analytical framework is available from the corresponding author on reasonable request. The interview topic guide is presented in Additional file 2.
