# Supplementary material for: Approaches to sharing trial results with adult patients and the public: a qualitative interview and focus group study
Source: Trials. 2026 Aug 18;27:664. doi: 10.1186/s13063-026-09944-5 (PMC13761133; doi:10.1186/s13063-026-09944-5)
Supplement: Supplementary file 1 [file 13063_2026_9944_MOESM1_ESM.docx]

#### 1 Standards for Reporting Qualitative Research (SRQR)*

|  | | **Page/line no(s).** |  |
| --- | --- | --- | --- |
| **Title and abstract** |  | | |
| **Title** - Concise description of the nature and topic of the study Identifying the study as qualitative or indicating the approach (e.g., ethnography, grounded theory) or data collection methods (e.g., interview, focus group) is recommended | | 1 |  |
| **Abstract** - Summary of key elements of the study using the abstract format of the intended publication; typically includes background, purpose, methods, results, and conclusions | | 2 |  |
|  | |  |  |
| **Introduction** |  | | |
| **Problem formulation** - Description and significance of the problem/phenomenon studied; review of relevant theory and empirical work; problem statement | | 5-6 |  |
| **Purpose or research questio**n - Purpose of the study and specific objectives or questions | | 5-6 |  |
|  | |  |  |
| **Methods** |  | | |
| **Qualitative approach and research paradigm** - Qualitative approach (e.g., ethnography, grounded theory, case study, phenomenology, narrative research) and guiding theory if appropriate; identifying the research paradigm (e.g., postpositivist, constructivist/ interpretivist) is also recommended; rationale** | | 7 |  |
| **Researcher characteristics and reflexivity** - Researchers’ characteristics that may influence the research, including personal attributes, qualifications/experience, relationship with participants, assumptions, and/or presuppositions; potential or actual interaction between researchers’ characteristics and the research questions, approach, methods, results, and/or transferability | | 9-10 |  |
| **Context** - Setting/site and salient contextual factors; rationale** | | 7-8 |  |
| **Sampling strategy** - How and why research participants, documents, or events were selected; criteria for deciding when no further sampling was necessary (e.g., sampling saturation); rationale** | | 7-8 |  |
| **Ethical issues pertaining to human subjects** - Documentation of approval by an appropriate ethics review board and participant consent, or explanation for lack thereof; other confidentiality and data security issues | | 7 |  |
| **Data collection methods** - Types of data collected; details of data collection procedures including (as appropriate) start and stop dates of data collection and analysis, iterative process, triangulation of sources/methods, and modification of procedures in response to evolving study findings; rationale** | | 8 |  |
| **Data collection instruments and technologies** - Description of instruments (e.g., interview guides, questionnaires) and devices (e.g., audio recorders) used for data collection; if/how the instrument(s) changed over the course of the study | | 8 |  |
| **Units of study** - Number and relevant characteristics of participants, documents, or events included in the study; level of participation (could be reported in results) | | 11-12 |  |
| **Data processing** - Methods for processing data prior to and during analysis, including transcription, data entry, data management and security, verification of data integrity, data coding, and anonymization/de-identification of excerpts | | 9 |  |
| **Data analysis** - Process by which inferences, themes, etc., were identified and developed, including the researchers involved in data analysis; usually references a specific paradigm or approach; rationale** | | 9 |  |
| **Techniques to enhance trustworthiness** - Techniques to enhance trustworthiness and credibility of data analysis (e.g., member checking, audit trail, triangulation); rationale** | | 9-10 |  |
|  | |  |  |
| **Results/findings** |  | | |
| **Synthesis and interpretation** - Main findings (e.g., interpretations, inferences, and themes); might include development of a theory or model, or integration with prior research or theory | | 11-26 |  |
| **Links to empirical data** - Evidence (e.g., quotes, field notes, text excerpts, photographs) to substantiate analytic findings | | 11-26 |  |
|  | |  |  |
| **Discussion** |  | | |
| **Integration with prior work, implications, transferability, and contribution(s) to the field -** Short summary of main findings; explanation of how findings and conclusions connect to, support, elaborate on, or challenge conclusions of earlier scholarship; discussion of scope of application/generalizability; identification of unique contribution(s) to scholarship in a discipline or field | | 27-29 |  |
| **Limitations** - Trustworthiness and limitations of findings | | 28 |  |
|  | |  |  |
| **Other** |  | | |
| **Conflicts of interest** - Potential sources of influence or perceived influence on study conduct and conclusions; how these were managed | | N/A |  |
| **Funding** - Sources of funding and other support; role of funders in data collection, interpretation, and reporting | | 30 |  |
|  | |  |  |
| *The authors created the SRQR by searching the literature to identify guidelines, reporting standards, and critical appraisal criteria for qualitative research; reviewing the reference lists of retrieved sources; and contacting experts to gain feedback. The SRQR aims to improve the transparency of all aspects of qualitative research by providing clear standards for reporting qualitative research. | |  |  |
|  | |  |  |
| **The rationale should briefly discuss the justification for choosing that theory, approach, method, or technique rather than other options available, the assumptions and limitations implicit in those choices, and how those choices influence study conclusions and transferability. As appropriate, the rationale for several items might be discussed together. | |  |  |
|  | |  |  |
|  | |  |  |
|  | |  |  |

#### 2 **Recruitment pathways for study participants**

| **Recruitment Pathway** | **Details** |
| --- | --- |
| **Group 1: Trial Professionals** |  |
| ***Trial Networks*** | - UK Trial Manager’s Network (UKTMN)  - UK Clinical Research Collaboration (UKCRC) Registered Clinical Trials Unit  - MRC-NIHR Trials Methodology Research Partnership (TMRP) groups  - NIHR Oxford Biomedical Research Centre (BRC)  - NIHR Centre for Engagement & Dissemination |
| ***Local Trial Units*** | - Oxford Clinical Trials Research Unit (OCTRU)  - National Perinatal Epidemiology Unit (NPEU)  - Diabetes Trials Unit (DTU)  - Clinical Trial Service Unit (CTSU)  - Surgical Intervention Trials Unit (SITU)  - Nottingham Biomedical Research Centre PPI network |
| ***Known Contacts*** | - Cambridge Clinical Trials Unit  - Hertfordshire research group  - Researcher and supervisor’s personal networks |
| ***University Network*** | - NDORMS Departmental Bulletin |
| **Group 2: Patient representatives and member of the public** |  |
| ***Diversity Networks website advertisement*** | - Oxford BRC, Diversity in Research Group ([link](https://oxfordbrc.nihr.ac.uk/pair/join-our-study-on-how-to-share-trial-results-with-adult-patients-and-the-public/))  - People in Research Group ([link](https://www.peopleinresearch.org/opportunity/join-our-study-on-how-to-share-trial-results-with-adult-patients-and-the-public/)) |
| ***Social Media*** | - #OxfordPPI Twitter  - Researcher’s personal Twitter accounts |
| ***Religious and Community Groups*** | - Oxford Islamic Society Community of Women |
| ***Patient and Public Involvement (PPI) Networks*** | - Local PPI Groups  - NDORMS OpenArms PPI group  - Be Part of Research-NIHR |
| ***Snowball Sampling*** | - Personal contacts |

#### 3 Interview topic guide

Group 1 topic guide:

**Consent:**

**Q: Have you had sufficient time to read and understand the information that was sent to you?**

*Prompts:*

1. Before we start, do you have any questions about this research?

2. is there something you do not understand?

**[Start recording]**

**Q. Do you agree/consent that you understand what the study involves, are happy to participate and are happy for this interview to be audio recorded?**

Your study ID number is: XX

**Involvement in trials:**

**Q. Any current or previous experiences or involvement in clinical trials?**

***Prompt***

a. What was/is your role?

b. How many years have you been involved in a trial?

**Q. Are you sharing or planning to share results with patients and the public at the end of your trial? / Have you previously shared results…?**

**A. If yes:**

A1. **Please could you tell me why you are sharing the trial results?**

A2. **What does sharing results with patients and the public mean to you in context of your trial?**

A3. **How are you sharing the results with them?**

***Prompt***

a. When did you plan to share results of your trial? Did you plan it from the beginning?

b. Talk me through the process.

A4. **What does your end product look like?**

***Prompt:***

a. What are the contents of your report?

b. What are the mandatory/optional items?

c. How long is your report? (word count etc.)

d. Who prepared the report from your team? In your opinion, who is primarily responsible for preparing the result report?

e. Is this the only way you shared the results? Would you plan to include any interactive elements in the report (e.g., Q&A sessions, forums)?

f. What is your planned timeline for sharing the results?

A5. **How did you decide what to write? How and why did you decide on those strategies?**

***Prompt***

a. Are you following any guidance?

b. Did you receive any plain language training

A6. **How will the participants receive the report?**

***Prompt***

a. What are some of the formats and channels you have used or are planning to use to share results with patients and the public? And why?

b. For example, will you be using written reports or infographics or videos or podcasts or telephone calls (audio format)?

c. Did the participants opt to receive the results?

A7. **What were your priorities when presenting the information in the report?**

*Prompt*

a. *How would you make sure that patients and the public will understand the information?*

A8. **What do you think about involving patients and the public in the process of sharing results?**

***Prompt***

a. Can you share some ways your trial involved patients and the public in the process of sharing results at the end of the trial?

A9. **What is your opinion on the resources needed to complete this process?**

***Prompt***

a. Would you plan a budget for this process?

A10. **Have you faced or are you anticipating any challenges in facilitating the sharing results process?**

A11. **What do you think would be the impact on patients and the public upon receiving the result?**

***Prompt***

a. emotional consequence/ improved understanding of the trial

**B. If no,**

B1. **What is your opinion on sharing trial results to patients and the public at the end of a trial?**

B2. **Can you share why your trial is not planning to share trial results?**

B3. **Hypothetically, if you were sharing the results with patients and the public, how would you structure your report?**

***Prompt:***

a. What mandatory/optional items would you consider to be included in a report of trial results for patients and the public?

b. Would you include visuals?

c. How long do you think the report should be?

d. Would you want to include any interactive elements in the report (e.g., Q&A sessions, forums)?

e. When do you think it is appropriate to release trial results reports to the public?

f. In your opinion, who should be preparing the result report?

B4. **How will you decide on those strategies? What are your priorities?**

***Prompt***

c. Will you follow any guidance that you know of on sharing results with patients and the public?

B5. **How will the participants receive the report?**

***Prompt***

a. What format or channel would you think to use for dissemination?

b. For example, will you use written reports, infographics, videos, or summary text?

g. What is your planned timeline for sharing the results?

B6. **What will be your priorities when presenting any information in the report?**

***Prompt***

b. *How would you make sure that patients and the public understand the information presented?*

B7. **Do you anticipate any potential challenges in facilitating this process?**

B8. **What do you think would be the impact on patients and the public upon receiving the result?**

***Prompt***

a. emotional consequence/ improved understanding of the trial

B9. **Would you engage patients and the public in preparing the result report?**

B10. **What is your opinion on the resources needed to complete this process?**

***Prompt***

a. Would you plan a budget for this process?

**Focus group:**

We would also do a focus group discussion with interested participants on this topic. In a focus group discussion, members discuss a given topic in depth, sharing their opinions over a course of interaction with others. This process is conducted by a moderator.

**Q. Would you be interested in joining this focus group discussion?**

If yes, can I contact you to arrange the interview? Would you prefer to do the meeting online or face-to-face?

**Closing:**

We are at the end of the interview now.

**Q. Would you like to share any additional thoughts or considerations that I have not covered?**

**Demographic Information:**

I would now like to collect some brief demographic information from you.

**Q. How would you describe your ethnicity?**

**Q. Are you ok with sharing your age? If not, could you give me an age range?**

**18-25, 26-35, 36-45, 46-55, 56-65, >65**

**Q. How would you describe your gender?**

**a. Male, Female, Prefer to identify as_____, Prefer not to say**

**Q. What is your Current job title?**

**Q. What is your current residing city? Would you be ok with sharing your postcode?**

**Q. Would you like to receive a summary of the findings of this study when it is finished? [If so, would it be OK if I sent that to you via email?]**

**Q. Would you be happy for us to keep your contact details for any future research associated with this DPhil, for example to contribute towards a focus group to feedback on any guidance that is developed?**

Thank you again for your time today.

**[stop recording]**

Group 2 topic guide:

**Consent:**

**Q: Have you had enough time to read and understand the information that was sent to you?**

***Prompts:***

1. Before we start, do you have any questions about this research?

2. is there something you do not understand?

**[Start recording]**

**Q. Do you agree/consent that you understand what the study involves and are happy to take part?**

**Q, Are you happy for me to record this interview?**

Your study ID number is: XX

[Participant to give verbal consent. If not, do not proceed]

**Transition to interview questions**

**Have you ever been or are you currently involved in a trial as a participant/patient/PPI?**

**A. If yes, were results shared with you or is there a plan to share results with you?**

A1. Could you talk me through the process of how you received the results?

A2. When did you receive the results?

A3. What was the format?

A4. What did you like about the report?

A5. What was difficult to understand?

A6. Did you opt to receive the results?

A7. Was there a follow-up session/resources provided to help with understanding the results?

A8. What would you have liked to be different?

**B. If no, Imagine, you were a participant in a trial.**

B1. **Do you think researchers should share the final trial results with you and the other people who took part in the trial and with the public?**

***Prompt***

a. Could you share some of the reasons why you think it is important/unimportant for researchers to share results with patients and the public?

b. Do you think study participants and the public would benefit from receiving the results?

B2. **What items would you like to see in the trial results report? And why?**

***Prompt***

a. What results or other information would be the most meaningful to include? Why?

b. What other things could the researchers include?

c. How long should the report be?

B3. **How would you like to receive the report?**

a. (e.g., email, postal mail, online platforms)? Why?

b. When would you like to receive it?

c. Would you like supporting material to help with your understanding?

d. Would you like an interactive element in the result sharing process such as Q&A session with the researchers?

B4. **How would you like the researcher to present the information? And why is that?**

***Prompt***

a. What would help with understanding and usability, e.g., using written reports?

b. Should the report include audio visuals? Such as pictures/graphs/, infographics /videos

c. Would you prefer an audio format? Such as a telephone call or a podcast

B5. **Do you have any previous experience with receiving such reports?**

B6. **What sort of impact do you think hearing the results will have on those who took part and their families?**

(e.g. Emotional consequences), Impact future participation in trials

B7. **If your native language is not English, would you like to receive a translated version of the results report in addition with the main report?**

B8. **What other recommendations do you have for researchers who may be planning to share results that we have not already discussed?**

**Focus group:**

We would also do one focus group discussion with interested participants on this topic. In a focus group discussion, instead of individual interviews, members get a chance to have an open discussion with each other and voice their opinions over a course of interaction with others. This will take about an hour of your time.

**Q. Would you be interested in joining this focus group discussion?**

**If yes, can I contact you to arrange the interview? Would you prefer to do the meeting online or face-to-face?**

**Closing:**

We are at the end of the interview now.

**Q. Would you like to share any additional thoughts or considerations that I have not covered?**

**Demographic Information:**

We want this study to be inclusive and recruit participants from diverse backgrounds. I would now like to learn a little bit about you, it is fine to say, ‘I’d rather not answer that’ to any of the questions.

**Q. How would you describe your ethnicity?**

**Q. Are you ok with sharing your age? If not, please could you give me an age range:**

**a. 18-25, 26-35, 36-45, 46-55, 56-65, >65**

**Q. How would you describe your gender?**

**a. Male, Female, Prefer to identify as_____, Prefer not to say**

**Q. What is your native language?**

**Q. Any current or previous experiences or involvement in clinical trials?**

**Q. Which city (? Or county) do you live in?** **Would you be ok with sharing your postcode?**

**Q. Please could you tell me about your family.**

**Q. Would you like to receive a summary of the findings of this study when it is finished? [If so, would it be OK if I sent that to you via email?]**

**Q. Would you be happy for us to keep your contact details for any future research associated with this DPhil, for example to contribute towards a focus group to feedback on any guidance that is developed?**

Thank you again for your time today.

**[stop recording]**

Focus group topic guide

**Introduction**

Thank you for attending the meeting today. This will be a focus group discussion, which means we will discuss our topic of trial result reports by interacting with each other. I will be the moderator conducting this process.

The discussion should last no longer than one hour.

All information collected will be confidential and participants names will not be disclosed in the final report. Data will be de-identified and stored confidentially in line with the General Data Protection Regulation (GDPR) and Data Protection Act 2018.

Topic:

1. **Preliminary results from the interviews**
2. **Invite comments on the results**
3. **What does sharing trial results with patients and the public mean to you?**
4. **How can we improve the result sharing practice?**
5. **Would you like to add something?**
6. **How should the report include visuals/audio (podcasts)?**
7. **Mention unusual items recorded during in-depth interviews and ask for opinions.**
8. **Would you like a follow-up Q&A session for the report?**
9. **Is there anything else you would like to share?**
10. **Does anyone want to share their experience of receiving such results?**

#### 4 Participant characteristics

***Figure: Geographic distribution of participants***

**Table: Summary table of participants characteristics**

| **Category** | **Group 1: Researchers working in trials (n=15)** | **Group 2: Members of the public and patient representatives (n=14)** | **Overall (n=29)** |
| --- | --- | --- | --- |
| **Sex** | | | |
| Female | 11 (73.3%) | 10 (71.4%) | 21 (72.4%) |
| Male | 4 (26.7%) | 4 (28.6%) | 8 (27.6%) |
| **Age (years)** | | |  |
| 21-30 | 3 (20%) | 3 (21.4%) | 6 (20.7%) |
| 31-40 | 1 (6.7%) | 3 (21.4%) | 4 (13.8%) |
| 41-50 | 7 (46.7%) | 0 (0%) | 7 (24.1%) |
| 51-60 | 4 (26.7%) | 2 (14.3%) | 6 (20.7%) |
| 61-70 | 0 (0%) | 2 (14.3%) | 2 (6.9%) |
| 71-80 | 0 (0%) | 4 (28.6%) | 4 (13.8%) |
| **Ethnicity** | | | |
| White British | 12 (80%); English (n=11), Scottish (n=1) | 10 (71.4%); English-9, Welsh-1 | 22 (75.9%) |
| White American | 1 (6.7%) | 0 (0%) | 1 (3.4%) |
| White other Turkish | 1 (6.7%) | 0 (0%) | 1 (3.4%) |
| Asian Indian | 1 (6.7%) | 1 (7.1%) | 2 (6.9%) |
| Asian Pakistani | 0 (0%) | 2 (14.3%) | 2 (6.9%) |
| Black African | 0 (0%) | 1 (7.1%) | 1 (3.4%) |
